# Upregulation of SLAMF3 on human T cells is induced by palmitic acid through the STAT5-PI3K/Akt pathway and features the chronic inflammatory profiles of type 2 diabetes

**DOI:** 10.1038/s41419-019-1791-y

**Published:** 2019-07-22

**Authors:** Tong Zhou, Guixia Wang, Yanan Lyu, Lei Wang, Siyao Zuo, Jun Zou, Lin Sun, Wenjie Zhao, Chang Shu, Yong-Guang Yang, Zheng Hu

**Affiliations:** 1 Key Laboratory of Organ Regeneration & Transplantation of Ministry of Education, The First Hospital of Jilin University, Changchun, 130061 China; 2 Department of Endocrinology and Metabolism, the First Hospital of Jilin University, Changchun, 130061 China; 3 National-Local Joint Engineering Laboratory of Animal Models for Human Diseases, Changchun, 130061 China; 4 International Center of Future Science, Jilin University, Changchun, 130012 China

**Keywords:** Lymphocyte activation, Chronic inflammation

## Abstract

Metabolic stress-induced low-grade chronic inflammation plays an important role in the development of insulin-resistance and type 2 diabetes (T2D). Emerging evidence highlights the importance of directly elucidating T-cell activation under the obesity-induced metabolic stress condition, as T cells primed under such conditions were found to acquire a unique phenotype and function. Herein, we found a significant upregulation of signaling lymphocytic activation molecule family member 3 (SLAMF3) expression on T cells from T2D patients compared to those of healthy controls. Importantly, SLAMF3 upregulation was associated with an increased ability to produce proinflammatory cytokines. Significantly increased SLAMF3 expression was seen in T2D patient T cells that produce IFN-γ or IL-17 upon short (4-h) stimulation, compared to non-cytokine-producing T cells. In line with this finding, SLAMF3^high^ T cells were significantly more sensitive than SLAMF3^low^ T cells to TCR stimulation with anti-CD3/CD28 antibodies. Furthermore, treatment with palmitic acid (PA) led to significant upregulation of SLAMF3 on human T cells primed by anti-CD3/CD28 antibodies and on Jurkat cells, a human T-cell line. RNA sequencing revealed strong activation of the PI3K/Akt signaling pathway in T cells that were primed with PA. Further mechanistic studies showed that inhibition of PI3K/Akt signaling, or its upstream mediator STAT5 can prevent PA-induced SLAMF3 upregulation on T cells. These results indicate that SLAMF3 upregulation is associated with T-cell activation and cytokine production in T2D patients, and suggest that elevated saturated fatty acids in T2D patients may induce SLAMF3 upregulation on T cells via activation of the STAT5-PI3K/Akt signaling pathway.

## Introduction

As a metabolic disorder characterized by insulin resistance and hyperglycemia, type 2 diabetes (T2D) is also associated with low-grade chronic inflammation that may result in a series of diabetes-related complications, including cardiovascular diseases, nephropathy, retinopathy, and increased risk for bacterial infection^1^. T2D patients have been reported to exhibit a decrease in NK, Th2, and regulatory T cells, an increase in Th1 and Th17 cells^2,3^, and a higher production of inflammatory cytokines such as IFN-γ, IL-17, and TNF-α^4^. Previous studies revealed that T-cell activation and infiltration into obese adipose tissues play an important role in the initiation and progression of adipose tissue inflammation, by recruiting macrophages and inducing obesity-associated insulin resistance^5,6^. In line with these findings, immunotherapy using T-cell-targeted antibody was effective in reversing high-fat diet (HFD)-induced insulin resistance^7^.

An increase in nonesterified fatty acids (NEFAs) is involved in obesity-associated insulin resistance and linked to T2DM^8^. It has been reported that palmitic acid (PA), a major component of the saturated NEFAs in HFD, as well as in the plasma of individuals with obesity and T2DM, is a detrimental factor that promotes inflammation and insulin resistance^8^. PA could induce inflammatory injury through an agonist membrane receptor, such as Toll-like receptor 4 (TLR4)^9^, and stimulate T cells to produce reactive oxygen species (ROS) and cytokines^10^. A recent report demonstrated that incubation of mouse CD4 T cells with PA induces CD4 T cells to acquire a CD44^hi^CCR7^lo^CD62L^lo^CXCR3^+^LFA1^+^ proinflammatory phenotype^11^. Although the exact mechanisms are not understood, these reports indicate that PA plays a critical role in inducing inflammatory profiles in T2D.

The signaling lymphocytic activation molecule family member 3 (SLAMF3) is a member of the SLAMF receptors that are widely expressed on lymphohematopoietic cells and regulate immune function through homophilic interactions^12^. T cells harvested from SLAMF3^−/−^ mice exhibit poor proliferation and little IL-2 production in response to suboptimal stimulation by anti-CD3 antibody in vitro, indicating that SLAMF3 signaling is involved in T-cell action^13^. Similarly, SLAMF3 has been found to be upregulated in patients with autoimmune disorders, such as systemic lupus erythematosus, supporting its role in promoting T-cell responses^14,15^. However, SLAMF3-deficient mice develop spontaneous systemic autoimmunity, suggesting that SLAMF3 may also serve as a receptor contributing to immune tolerance^16^. The reasons for these contradictory observations remain unclear. In this study, we found that SLAMF3 is upregulated on T cells from T2D patients. Importantly, increased SLAMF3 expression on T cells was associated with improved potential to produce proinflammatory cytokines and enhanced responses to TCR stimulation. Further mechanistic studies showed that exposure of T cells to PA could significantly upregulate SLAMF3 expression through activation of the STAT5-PI3K/Akt signaling pathway, providing direct evidence linking chronic inflammation with elevated plasma NEFAs in T2D patients.

## Materials and methods

### Human subjects

Subjects diagnosed with T2DM (*n* = 76) and healthy control subjects (HC; *n* = 74) were recruited from the Department of Endocrinology and Metabolism and the physical examination center of the First Hospital of Jilin University. Exclusions were severe complications (such as renal failure, severe microvascular disease, severe macrovascular disease, or blindness), chronic or common infections (HBV, HCV, influenza, and fever), cancer, long-term use of immunosuppressive agents, or merging with other autoimmune disease. Umbilical cord blood was collected from the Department of Obstetrics of the First Hospital of Jilin University with informed consent for human T-cell function measurement. Protocols involved in the use of human samples were approved by the Institutional Review Board, and all of the experiments were performed in accordance with the protocols.

### Flow cytometric assays

Peripheral blood mononuclear cells (PBMCs) were isolated from heparinized whole blood using density gradient centrifugation from T2D patients or HCs, and stained with various combinations of the following fluorescence conjugated antibodies: anti human-CD3, CD4, CD8, CD25, CD229 (SLAMF3), IL-17, and IFN-γ (BD Pharmigen). BD Cytofix/Cytoperm Fixation and Permeabilization Kits (BD) were used for intracellular staining assays to detect cytokine production of T cells from T2D patients that were stimulated for 4 h with Leukocyte Activation Cocktail (BD GolgiPlug™, including PMA, ionomycin, and brefeldin A). Flow cytometric measurement was performed with a FACS Fortessa (BD Biosciences) and the data were analyzed by FlowJo (version 10).

### MACS based cell sorting

Mononuclear cells were prepared from umbilical cord blood by density gradient centrifugation using Histopaque (1.077, SIGMA), and then sorted for CD4^+^ cells by MACS positive selection. Briefly, the cells were incubated firstly with biotin-conjugated antihuman CD4 antibody (BD) and then streptavidin-conjugated Micro-beads (Miltenyi Biotec) after washing. CD4^+^ T cells were collected from the cells retained in LS column under magnetic field. The purity of sorted cells was more than 97% as confirmed by flow cytometric analysis.

### Human T-cell expansion assays

CD3^+^CD4^+^CD25^−^SLAMF3^high^ (SLAMF3^high^ CD4 T cells) and CD3^+^CD4^+^CD25^−^SLAMF3^low^ (SLAMF3^low^ CD4 T cells) were purified by FACS cell sorting (BD Influx) and the purity was confirmed by flow cytometric assays (>97% purity). SLAMF3^high^ or SLAMF3^low^ CD4 T cells were incubated in the presence of precoated antihuman CD3 and soluble antihuman CD28 antibodies at the indicated concentrations in 37 °C incubator with 5% CO_2_. Seven days later, the cell expansion was measured by flow cytometric analysis and the images under microscope.

### Measuring SLAMF3 expression on human T cells under different conditions

CD4^+^ cord blood T cells were stimulated with CD3/CD28 antibodies (as detailed above) for 72 h and analyzed for SLAMF3 expression by flow cytometry analysis. We also measured SLAMF3 expression on human T cell Jurkat cells (pass mycoplasma contamination test) after incubated for 72 h under similar conditions without addition of antihuman CD3/CD28 antibodies in the absence or presence of PA (0.1, 0.15, or 0.3 mM), PI3K inhibitor (LY294002, sigma), and/or STAT5 inhibitor (sc-355979/Si, SCBT).

### RNA sequence

CD4^+^ cord blood T cells were stimulated with CD3/CD28 antibodies in the absence or presence of 0.3 mM PA for 72 h in 37 °C incubator with 5% CO_2_. After confirming SLAMF3 expression by flow cytometry analysis, total RNA was extracted from the cells by TRIzol (Invitrogen) and RNA sequencing was performed by Novogene. Differentially expressed genes between CD4 T cells that were stimulated in the absence or presence of PA were identified using the significance of digital gene expression profiles^17^. Prior to differential gene expression analysis, for each sequenced library, the read counts were adjusted by edgeR program package through one scaling normalized factor. Differential expression analysis of two conditions was performed using the edgeR package^18^. The *p* values were adjusted using the Benjamini and Hochberg method. Corrected *p* value of 0.05 and absolute foldchange of two were set as the threshold for significantly differential expression. Kyoto encyclopedia of genes and genomes (KEGG) pathways or Disease Ontology (DO) terms were considered. The method of calculating the *p* value was performed traditionally^19^. Then, the enriched significance *p* value was adjusted using the Benjamini and Hochberg algorithm^20^. Finally, KEGG pathways or DO terms with adjusted *p* values < 0.05 and including at least two differentially expressed genes were considered.

### Statistical analysis

All analyses were performed with GraphPad Prism version 6. Control and experimental results were compared with the nonparametric Wilcoxon/Kruskal–Wallis or the paired two-tailed Student’s *t*-test. For all analysis, statistical significance was reported as follows: *p < 0.05; **p < 0.01; ***p < 0.001; ns, no significance.

## Results

### SLAMF3 expression is upregulated on T cells from T2D patients

Seventy-six T2D participants that met the inclusion criteria and 74 healthy controls (HCs) were enrolled in the study (Table 1). The mean age and average body mass index (BMI) were 52.15 years and 25.53 kg/m^2^ for T2D patients, and 49.37 years and 25.14 kg/m^2^ for HCs. T2D patients had an average HbA1c level of 8.81% and significantly higher fasting blood glucose (FBG; 8.99 mmol/L) than HCs (5.23 ± 0.47 mmol/L). T2D patients also exhibited significantly increased triglyceride (TG; 2.85 ± 2.41 mmol/L) and decreased high-density lipoprotein cholesterol (HDL-C; 1.09 ± 0.29 mmol/L) compared to HCs.

**Table 1:** Baseline clinical characteristics and the results of biochemical tests

	HC	T2DM
Total N	74	76
Age	49.37 ± 11.34	52.15 ± 9.11
Male	47	49
Female	27	27
BMI (kg/m^2^)	25.47 ± 3.72	25.14 ± 3.42
FBG (mmol/L)	5.23 ± 0.47	8.99 ± 2.94^***^
HbA1c (%)	–	8.81 ± 1.88
TC (mmol/L)	5.15 ± 0.94	4.82 ± 1.29
TG (mmol/L)	1.68 ± 0.98	2.85 ± 2.41^***^
LDL-C (mmol/L)	3.08 ± 0.70	2.84 ± 0.79
HDL-C (mmol/L)	1.47 ± 0.37	1.09 ± 0.29^***^

Results are expressed as the mean ± SD

*BMI* body mass index, *FBG* fasting blood glucose, *TC* total cholesterol, *TG* triglyceride, *LDL-C* low-density lipoprotein cholesterol, *HDL-C* high-density lipoprotein cholesterol

**p < 0.01, ***p < 0.001

**Fig. 1:**
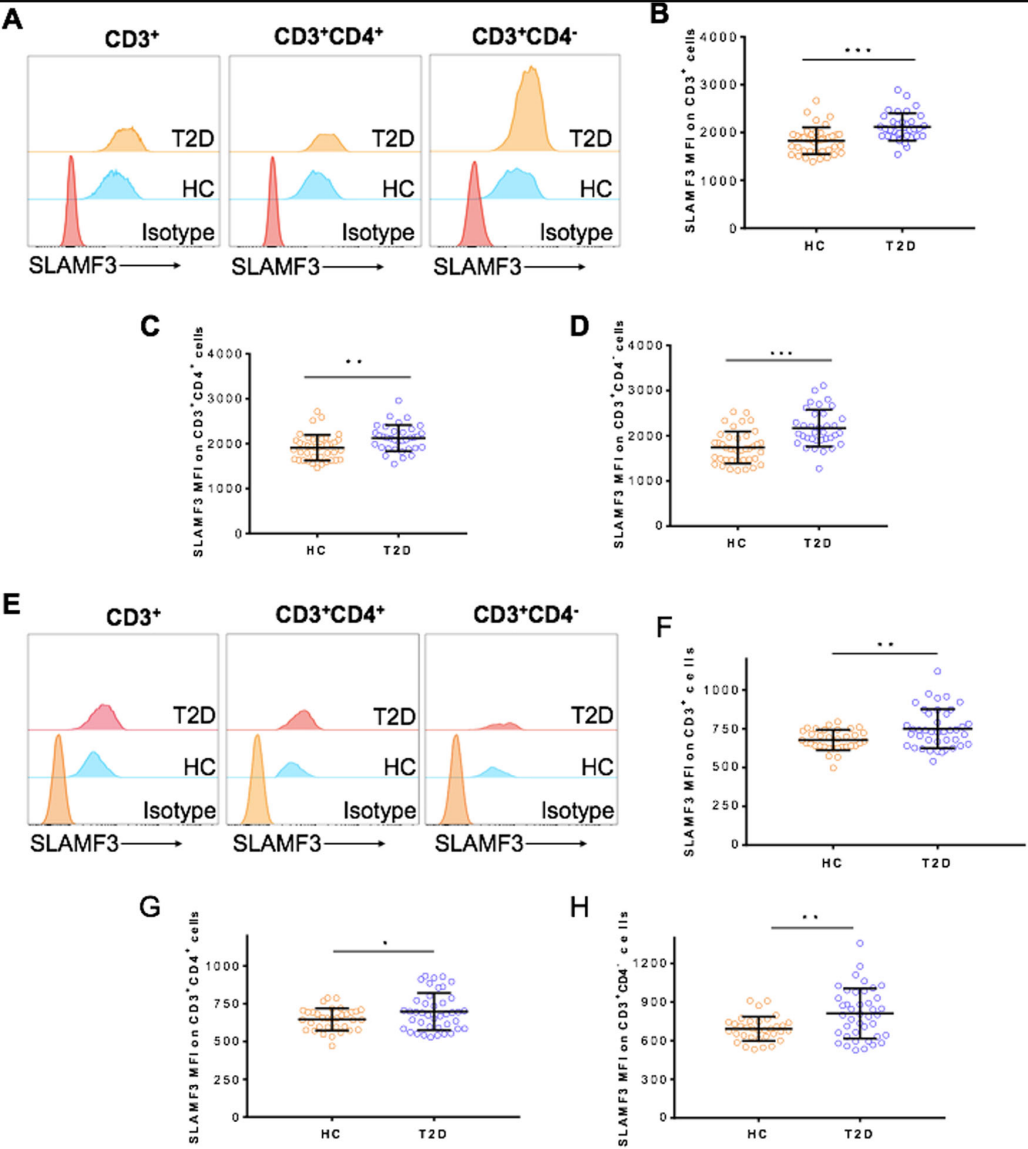
Elevation of SLAMF3 on the human T-cell surface in T2D patients. SLAMF3 expression on human T cells in the PBMCs of T2D patients (*n* = 76) and HCs (*n* = 74) were analyzed by flow cytometry, in which the cells were stained freshly for only cell surface markers (**a**–**d** *n* = 35 and 40 for T2D and HCs, respectively), or fixed/permeabilized for staining of both cell surface and intracellular proteins (**e**–**h** *n* = 41 and 34 for T2D and HCs, respectively). **a**, **e** Representative flow cytometric profiles of SLAMF3 in CD3^+^ T cells (left), CD3^+^CD4^+^ (middle), and CD3^+^CD4^−^ T cells (right) were shown. **b**–**d**, **f**–**h** Summarized results about the median fluorescent intensity (MFI, mean ± SD) of SLAMF3 on CD3^+^ T cells (**b**, **f**), CD3^+^CD4^+^ T cells (**c**, **g**) and CD3^+^CD4^−^ T cells (**d**, **h**) were shown. *p < 0.05; **p < 0.01; ***p < 0.001

PBMC samples collected from T2D patients and HCs were analyzed for T-cell subsets and phenotypes by flow cytometry. As shown in Fig. S1, T2D patients and HCs had a comparable level of total CD3^+^ T cells, but the level of CD3^+^CD4^+^ T cells was significantly increased in T2D patients compared to HCs. Interestingly, a notable change in T2D patients was the upregulated surface expression of SLAMF3 on T cells, including both CD4^+^ and CD4^−^ T-cell subsets (Fig. 1), suggesting a possible involvement of SLAMF3 signaling in altered immune responses in T2D patients.

### Higher surface SLAMF3 expression in T cells is associated with increased proinflammatory cytokine production and improved proliferative responses to anti-CD3/CD28

**Fig. 2:**
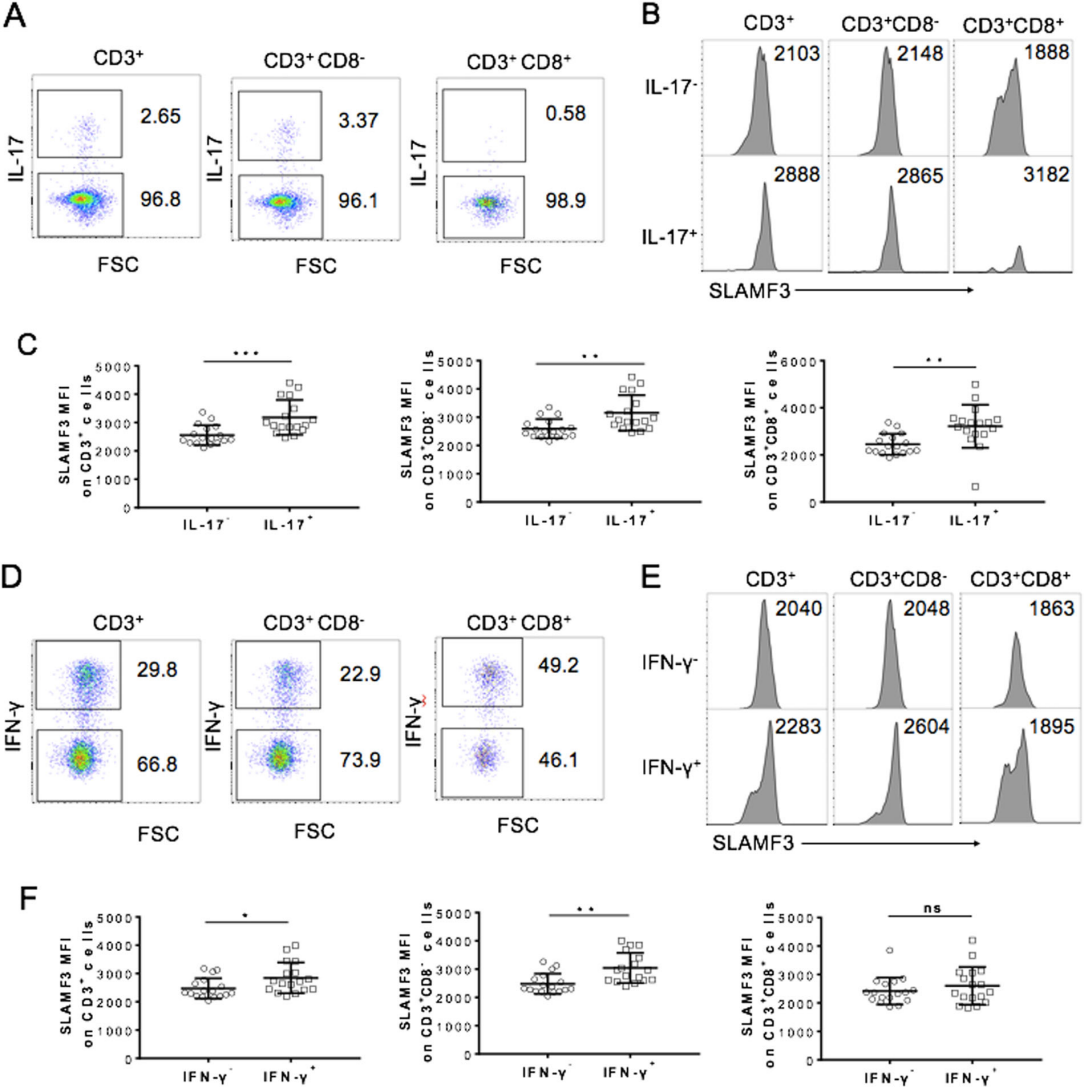
SLAMF3^high^ T cells possess strong capability to produce inflammatory cytokines. PBMCs (*n* = 17) of T2D patients were analyzed for inflammatory cytokine production by flow cytometry after 4-h stimulation by leukocyte activation cocktail. **a** Representative flow cytometric profiles about production of IL-17 by CD3^+^, CD3^+^CD8^−^, and CD3^+^CD8^+^ T cells were shown. **b** Representative flow cytometric profiles of SLAMF3 histogram in IL-17^−^ or IL-17^+^ in CD3^+^ (left), CD3^+^CD8^−^ (middle), and CD3^+^CD8^+^ T cells (right) were shown. **c** Summarized data about SLAMF3 MFI analyzed in IL-17 or IL-17^−^^+^ in CD3^+^ (left), CD3^+^CD8^−^ (middle), and CD3^+^CD8^+^ T cells (right) were shown. **d** Representative flow cytometric profiles about production of IFN-γ by CD3^+^, CD3^+^CD8^−^, and CD3^+^CD8^+^ T cells were shown. **e** Representative flow cytometric histogram profiles of SLAMF3 analyzed in IFN-γ^-^ or IFN-γ^+^ in CD3^+^, CD3^+^CD8^−^, and CD3^+^CD8^+^ T cells were shown. **f** Summarized data about SLAMF3 MFI analyzed in IFN-γ^−^ or IFN-γ^+^ in CD3^+^, CD3^+^CD8^−^, and CD3^+^CD8^+^ T cells were shown. ns no significant difference; *p < 0.05; **p < 0.01; ***p < 0.001

In T2D patients with chronic low-grade inflammation, a series of proinflammatory cytokines secreted by T cells (e.g., IFN-γ and IL-17) were found at increased levels^4^. Because SLAMF3 has been shown to work as a costimulatory molecule in the activation of human T cells^15^, we hypothesized that upregulated SLAMF3 expression on T cells may contribute to the persistent “low-grade inflammatory” status of T2D patients. To address this hypothesis, we compared the levels of SLAMF3 expression on T-cell subsets with different potentials to produce proinflammatory cytokines in T2D patients. PBMCs from T2D patients were stimulated for 4 h by PMA/ionomycin with brefeldin A, then T-cell production of IL-17 and IFN-γ and expression of SLAMF3 were measured by flow cytometry. Both IL-17- and IFN-γ-producing CD3^+^T cells showed significantly increased surface expression of SLAMF3 (Fig. 2). Further analysis revealed that both CD3^+^CD8^−^ and CD3^+^CD8^+^ IL-17-producing cells had significant upregulation of SLAMF3 (Fig. 2a–c). Meanwhile, IFN-γ-producing CD3^+^CD8^−^ T cells but not CD3^+^CD8^+^ T cells were found with SLAMF3 upregulation (Fig. 2d–f). These data indicate that higher surface SLAMF3 expression was associated with increased IFN-γ and IL-17 production in human T cells from T2D patients.

**Fig. 3:**
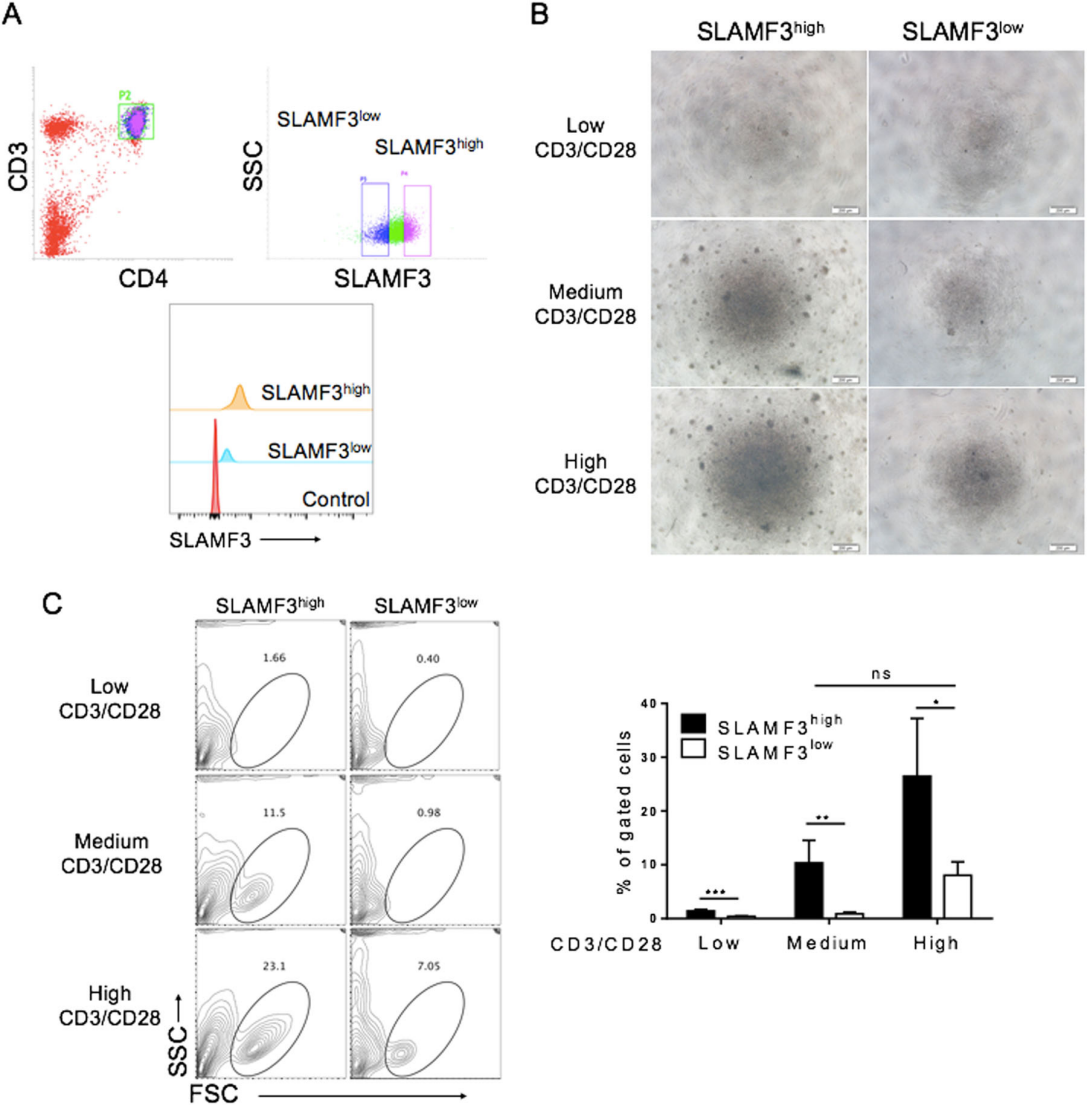
SLAMF3^high^ T cells are sensitive to CD3/CD28 stimulation. SLAMF3^high^ CD3^+^CD4^+^CD25^−^ T cells and SLAMF3^low^ CD3^+^CD4^+^CD25^−^ T cells were purified from cord blood mononuclear cells by FACS sorter, and stimulated with different concentrations of anti-CD3/anti-CD28 antibodies for 7 days in vitro (anti-CD3/anti-CD28 concentration: low 0.8 μg/ml/0.4 μg/ml, medium 2 μg/ml/1 μg/ml, and high 5 μg/ml/2.5 μg/ml). **a** Representative flow cytometric profiles about sorting strategy (up) and the purity of sorted SLAMF3^high^ or SLAMF3^low^ cells were shown. **b** Representative images of cell expansion 7 days after stimulation. Scale bars equal 200 μm. **c** Representative flow cytometric profiles (left) 7 days after stimulation were shown. Summarized data (right) about the ratios of live cells after expansion (*n* = 4 per group, mean ± SDs) based on flow cytometric analysis was shown. *p < 0.05; **p < 0.01; ***p < 0.001. (Data shown are results from a representative of three experiments)

To further understand the functional differences between T cells with different levels of SLAMF3 expression, we compared the proliferative response to anti-CD3/CD28 stimulation of purified SLAMF3^high^ (MFI = 677) and SLAMF3^low^ (MFI = 318) CD4^+^CD25^−^ T cells (Fig. 3a). SLAMF3^high^ CD4^+^CD25^−^ T cells showed significantly improved proliferation and expansion compared to SLAMF3^low^ CD4^+^CD25^−^ T cells at all three concentrations of anti-CD3/CD28 antibodies (referred to as low, medium, and high, respectively; Fig. 3). Robust expansion was only detected in SLAMF3^low^ CD4^+^CD25^−^ T cells that were stimulated with the high concentration of anti-CD3/CD28, whereas a comparable expansion was detected in SLAMF3^high^ CD4^+^CD25^−^ T cells stimulated with the medium concentration of anti-CD3/CD28 (Fig. 3b, c). These data indicate that SLAMF3^high^ T cells are more sensitive to TCR stimulation, providing a possible explanation for the observation that SLAMF3 upregulation was associated with increased potential to produce inflammatory cytokines in T cells from T2D patients.

### Palmitic acids upregulate SLAMF3 expression on human T cells

**Fig. 4:**
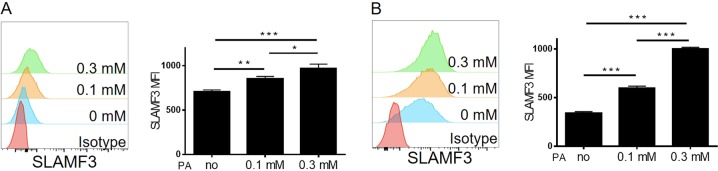
Palmitic acid treatment raises SLAMF3 on human T cells. **a** Purified human CD4 T cells were stimulated by 5 μg/ml precoated anti-CD3 and 5 μg/ml solute anti-CD28 antibodies in the presence of palmitic acid (0, 0.1, 0.3 mM, *n* = 3/each) for 3 days. Representative flow cytometric profiles (left) and summarized data (right; mean ± SDs) about SLAMF3 expression on T cells were shown. (Data shown are results from a representative of four experiments.) **b** Jurkat cells were cultured in different concentration of palmitic acid (0, 0.1, 0.3 mM, *n* = 3/each) for 3 days. Representative flow cytometric profiles (left) and summarized data (mean ± SDs, *n* = 3/each) about SLAMF3 expression were shown. (Data shown are results from a representative of three experiments)

As a “bad” fatty acid enriched in HFDs, PAs have been shown to induce T-cell activation and phenotypic alteration^5,10^. Therefore, we considered whether PAs could modulate SLAMF3 expression on human T cells. We measured SLAMF3 expression on T cells 3 days after stimulation with anti-CD3/CD28, in the absence or presence of PAs. T cells stimulated in the presence of PAs had significantly increased SLAMF3 expression on their surfaces, and the PA effect was dose dependent (Fig. 4a). Similar observations were made in human T-cell line Jurkat cells, in which a significant and dose-dependent upregulation of SLAMF3 expression was detected after culture with PAs, compared to culture without PAs (Fig. 4b).

### Palmitic acids upregulate SLAMF3 expression on human T cells through PI3K pathway activation

**Fig. 5:**
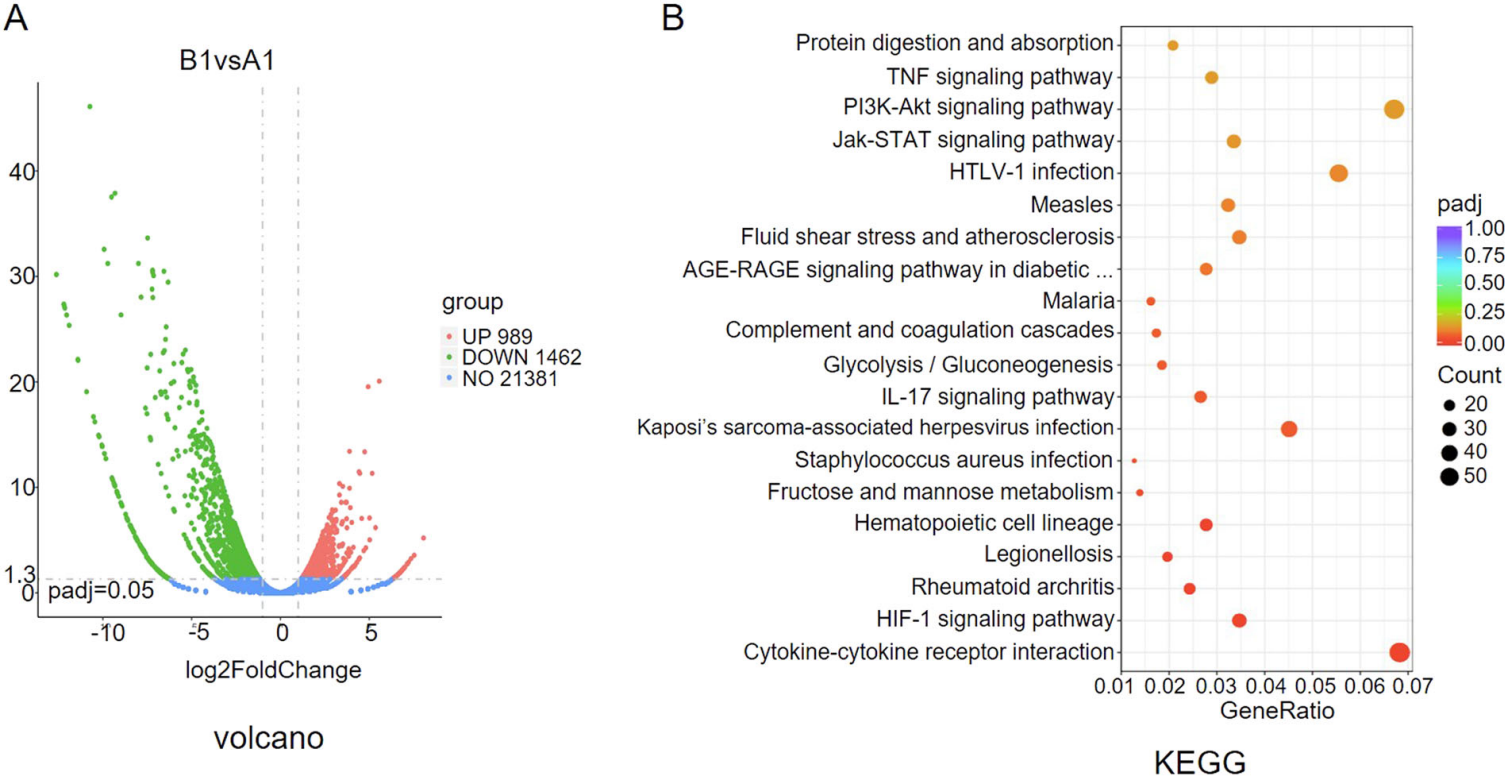
RNA-Seq analysis of the influences of palmitic acid to human CD4 T cells. Purified human CD4 T cells were stimulated with 5 μg/ml precoated anti-CD3 and 5 μg/ml solute anti-CD28 with or without 0.3 mM palmitic acid for 3 days, the RNA was extracted and send for RNA-Seq. **a** Volcano profiles of RNA sequence between the two groups. **b** Kyoto encyclopedia of genes and genomes (KEGG) pathway analysis of RNA sequence data. *p < 0.05, **p < 0.01, ***p < 0.001

To further understand the mechanisms by which PA treatment upregulates SLAMF3 expression on human T cells, RNA-Seq was performed on purified human CD3^+^CD4^+^ T cells that had been stimulated for 3 days with anti-CD3/CD28 without or with PAs (0.3 mM). Differential gene expression analysis revealed that 989 genes were upregulated and 1462 genes were downregulated in PA-treated human T cells (Fig. 5a), which is associated with T2D relevant complications including obesity, pancreas diseases, bacterial infection, coronary artery diseases, and so forth (Fig. S2). Moreover, PA treated human T cells showed activation of several signaling pathways, particularly those associated with viral infections and cytokine–cytokine receptor interactions (Fig. 5b), which is in agreement with the role of PA in T-cell activation (Fig. 4)^5,10^. The PI3K/Akt signaling pathway that was also markedly activated in T cells after PA treatment (Fig. 5b).

**Fig. 6:**
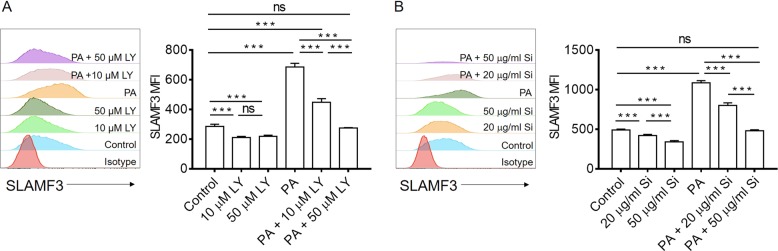
Inhibition of STAT5-PI3K/Akt pathway prevent the raising of SLAMF3 induced by palmitic acid on human T cells. **a** Jurkat T cells were cultured in the presence of palmitic acid (PA, 0 or 0.15 mM) with or without PI3K inhibitor, LY294002 (LY, 10 or 50 μM) for 3 days. Representative flow cytometric profiles (left) and summarized data (right; mean ± SDs, *n* = 4/each) about SLAMF3 expression were shown. (Data shown are results from a representative of three experiments.) **b** Jurkat T cells were cultured in the presence of palmitic acid (PA, 0 or 0.15 mM) with or without STAT5 inhibitor, sc-355979 (Si, 20 or 50 μg/ml) for 3 days. Representative flow cytometric profiles (left) and summarized data (right; mean ± SDs, *n* = 4/each) about SLAMF3 expression were shown. (Data shown are results from a representative of three experiments.) ***p < 0.001; ns no significant difference

In order to verify that the PI3K/Akt signaling pathway activation is involved in SLAMF3 upregulation in PA-treated T cells, we measured SLAMF3 expression on Jurkat cells that were cultured for 3 days in the absence or presence of PAs and/or the PI3K specific inhibitor, LY294002 (10 or 50 µM). Again, PA treatment significantly upregulated SLAMF3 expression on Jurkat cells, but this SLAMF3 upregulation on PA-treated cells was significantly and dose dependently inhibited by LY294002 (Fig. 6a). The effect of PAs on SLAMF3 expression in Jurkat cells was completely diminished by culture with LY294002 at the higher concentration 50 μM. Because the JAK/STAT pathway was also activated in PA treated T cells (Fig. 5b) and the JAK/STAT5 signals upstream of PI3K/Akt^21–23^, we measured SLAMF3 expression on Jurkat cells that were cultured with STAT5 inhibitor, sc-355979. As shown in Fig. 6b, culture with sc-355979 also significantly and dose dependently inhibited PA-induced SLAMF3 upregulation, with complete inhibition at 50 μg/ml. These data indicate that the STAT5-PI3K/Akt signaling pathway is important in mediating PA-induced upregulation of SLAMF3 on human T cells.

## Discussion

Low-grade chronic inflammation is considered a causative factor in the development of insulin resistance and T2D^1^. There is emerging evidence for obesity-induced immune responses. Recently, it was reported that memory T cells from hosts with or without obesity-induced metabolic stress are different in both phenotype and function^11^, highlighting the importance of characterizing biased T-cell activation under obesity conditions. Although there are still some controversies, previous studies provided strong evidence supporting the role of SLAMF3 in promoting T-cell activation^13–15,24^. Here we found that SLAMF3 is significantly upregulated on T cells from T2D patients; importantly, its upregulation on T cells was associated with increased potential to produce proinflammatory cytokines. In agreement with these observations made in T2D patients, human SLAMF3^high^ T cells were found to be more sensitive to TCR stimulation with anti-CD3/CD28 antibodies. Although our data cannot firmly demonstrate that SLAMF3 upregulation is necessary and/or sufficient for the observed chronic inflammatory phenotypes of T cells in T2D patients, these results indicate that SLAMF3 upregulation provides a marker for activated T cells that are the major source of T cell-derived proinflammatory cytokines in T2D patients.

PA is a common saturated fatty acid found in the plasma of individuals with obesity and T2DM and plays an important role in the induction of inflammation and the development insulin resistance^8^. PA was reported to interact with agonist membrane receptors (e.g., TLR4)^9^ and to induce ROS and cytokine production^10^. PA can also convert naïve T cells to a proinflammatory phenotype^11^. These studies raise the possibility that PA is an important factor driving T-cell activation and inflammation in T2D patients. In support of this possibility, we found that PA treatment significantly upregulated SLAMF3 expression on human T cells through induction of SLAMF3 gene transcripts (Fig. S3A). In addition, PA treatment raised inflammatory gene transcription, e.g., IFNG and IL17A in human T-cell line cells (Fig. S3B and C), while not affecting their proliferation (Fig. S4). Moreover, using humanized mice with a functional human immune system^25^, we revealed that HFD feeding induced not only an increase in the visceral adipose tissues, but also an elevation in serum total cholesterol (TC) and triglyceride (TG). Importantly, human T cells from HFD-treated mice showed a significantly upregulated SLAMF3 expression compared to those from HD-fed mice (Fig. S5). Together, these studies indicate that PA is an important factor causing chronic inflammation in T2D patients, and that PA-induced T-cell activation is associated with the upregulation of SLAMF3 surface expression.

A recent study demonstrated enhanced activation of a PI3K p110δ-Akt-dependent pathway in T cells that were primed in the presence of saturated fatty acid^11^. The PI3K/Akt pathway is known to be rapidly activated upon T-cell priming and to control transcriptional and metabolic programs that sustain cell activation^26^. In line with these observations, our RNA-Seq results showed that PA induced a markedly enhanced activation of the PI3K/Akt signaling pathway upon priming. Using the pan PI3K inhibitor LY294002, we further confirmed that PI3K/Akt signaling is required for PA-induced SLAMF3 upregulation on anti-CD3/CD28-stimulated T cells. JAK/STAT5 signals upstream of PI3K/Akt, acting in a linear pathway to modulate T-cell survival and function^21–23^. RNA-Seq data revealed that the JAK/STAT pathway was also activated in PA treated T cells, implying a possible role for STAT5 in driving PI3K/Akt activation in PA-treated T cells. In support of this possibility, we found that PA failed to upregulate SLAMF3 in T cells in the presence of STAT5 inhibitor. Thus, we propose that saturated fatty acid can induce SLAMF3 upregulation on T cells through activation of the JAK/STAT5-PI3K/Akt signaling pathway in T2D patients.

In summary, this study shows that SLAMF3 upregulation is associated with increased potential to produce inflammatory cytokines and to respond to TCR signaling of T cells in T2D patients. Furthermore, saturated fatty acid (e.g., PA) is an important factor that upregulates SLAMF3 expression on T cells, and this process requires activation of the STAT5-PI3K/Akt signaling pathway. These results provide insight into the immunopathogenesis of T2D and potential molecular targets for T2D therapies.

## Supplementary information

Supplemental material
