# Supplementary material for: Upregulation of SLAMF3 on human T cells is induced by palmitic acid through the STAT5-PI3K/Akt pathway and features the chronic inflammatory profiles of type 2 diabetes
Source: Cell Death Dis. 2019 Jul 22;10(8):559. doi: 10.1038/s41419-019-1791-y (PMC6646391; doi:10.1038/s41419-019-1791-y)
Supplement: Supplementary file 1 — Supplemental material [file 41419_2019_1791_MOESM1_ESM.docx]

**Upregulation of SLAMF3 on human T cells is induced by palmitic acid through the STAT5-PI3K/Akt pathway and features the chronic inflammatory profiles of type 2 diabetes**

Tong Zhou^1,2,3,4^, Guixia Wang^2*^, Yanan Lyu^1,3,4^, Lei Wang^1,2,3^, Siyao Zuo^1,3,4^, Jun Zou^1,3,4^, Lin Sun^1,2,3,4^, Wenjie Zhao^1,3,4^, Chang Shu^1^, Yong-Guang Yang^1,3,4^, Zheng Hu^1,3,4*^

**Supplementary Information**

Supplementary Materials and Methods

Supplementary Figures: 5.

**Mice and human samples**

NOD-Prkdc^em26Cd52^Il2rg^em26Cd22^/Nju (NOD/SCID IL2rg^-/-^ or NCG) mice were purchased from Nanjing Biomedical Research Institute of Nanjing University and were housed in a specific pathogen-free (SPF) micro-isolator environment. The mice were used in experiments at around 11 weeks of age. Discarded human fetal tissues with gestation of 17-21 weeks were obtained with informed consent. Humanized mice with human lymphocytic reconstitution were created by co-transplantation of human fetal thymic tissues (under kidney capsule) and fetal liver-derived CD34^+^ cells (2 × 10^5^/each, i.v.) into sublethally (2Gy)-irradiated NCG mice, as described before. Protocols involved in the use of human tissues and animals were reviewed and approved by Institutional Review Board and Institutional Animal Care and Use Committee of the First Hospital of Jilin University, and all of the experiments were performed in accordance with the protocols.

**High fat diet experiment**

Humanized mice were fed with normal diet (ND) or high fat diet (HFD, 60% fat, Research Diets, Ins, New Brunswich, USA) from week 5 after humanization. Human immune cell chimerism in mouse PBMCs was measured every 3 weeks by flow cytometry, using various combinations of the following mAbs: anti-human CD45, CD19, CD3, CD4, CD8, SLAMF3, anti-mouse CD45, Ter119 (purchased from Biolegend). After 12-week ND/HFD feeding, the animals were sacrificed, the visceral adipose tissues (VAT) from epididymal fat pads were weighted, and the level of serum total cholesterol (TC) and triglyceride (TG) were examined by lipid test kits (Nanjing Jiancheng Bioengineering Institute, China).

**Q-PCR**

Total RNAs were extracted from the samples using Trizol (Invitrogen), and then reverse-transcribed using HiScript III RT SuperMix kit (Vazyme Biotech Co., Ltd, Nanjing, China) according to manufacturer’s instructions. The threshold cycle (Ct) value of each sample was determined using ChamQ Universal SYBR qPCR Master Mix (Vazyme Biotech Co.,Ltd, Nanjing, China) in ABI 7300 Real-Time PCR System (Applied Biosystems, Foster City, CA). Relative mRNA transcripts of each target gene in PA treated samples were normalized to the corresponding ones in untreated sample. Q-PCR primer pairs were purchased from XYbiotech (*LY9/SLAMF3*, Cat: S350747; *IFNG*, Cat: DHS111004; *IL17A*, Cat: DHS376792, XYbiotech, China).

**Statistical analysis**

The level of significant differences in group means was determined by the Student’s t-test. All statistical analysis was performed using Prism 8 (GraphPad Software). Statistical significance was determined by using one-way analysis of variance (ANOVA) or Student's t-test. * *P* < 0.05 was considered significant.

**Figure S1. Human T cell combination in T2D and HC subjects.** Summarized data about the ratios of CD3^+^ and CD3^+^CD4^+^ T cells in the PBMCs of T2D and HC subjects (mean±SDs; n_DM_=76, n_HC_=74) are shown. **, *P*<0.01; ns, no significant difference.

**Figure S2. Disease oncology analysis for palmitic acid treated human CD4 T cells by RNA-Seq.** Purified human CD4 T cells were stimulated with 5 μg/ml pre-coated anti-CD3 and 5 μg/ml solute anti-CD28 with or without 0.3 mM palmitic acid for 3 days, the RNA was extracted and send for RNA-Seq. Disease Ontology (DO) analysis of RNA sequence data was shown.

**Figure S3.** PA treatment raises *SLAMF3*, *IFNG* and *IL17A* transcripts in Jurkat cells. Q-PCR analysis of *SLAMF3* (left), *IFNG* (middle) and *IL17A* (right) gene expression (mean±SD) in Jurkat cells treated with (0.3 mM, n=3) or w/o PA (UT, n=3) was shown. *, *P*<0.05; ***, *P*<0.001.

**Figure S4.** PA treatment does not influence Jurkat cell proliferation. CFSE labeled Jurkat cells were cultured in the complete 1640 medium with (n=3) or w/o (n=3) PA for 2 days. Representative flow cytometric profiles and CFSE MFI values (mean±SD) were shown. ns, no significant difference.

**Figure S5.** Upregulation of SLAMF3 expression on human T cells in humanized mice after HFD feeding. From week 5 after humanization, the humanized mice were fed with HFD (n=4) or ND (n=4) for 12 weeks. (A) The ratios (mean±SEM) of human CD45^+^ cells (left), CD3^+^ T cells (middle) and CD19^+^ B cells (right) in the PBMCs of humanized mice at indicated time after HFD/ND feeding were shown. (B) The VAT weights (left), levels of serum TC (middle) and TG (right) of humanized mice were recorded. (C). The chimerism of human CD45^+^ cells (left), T cells (middle) and SLAMF3 expression on human T cells in PBMCs 12 weeks after HFD/ND feeding were shown. *, *P*<0.05; **, *P*<0.01; ***, *P*<0.001; ns, no significant.
